# Mind evolutionary algorithm optimization in the prediction of satellite clock bias using the back propagation neural network

**DOI:** 10.1038/s41598-023-28855-y

**Published:** 2023-02-06

**Authors:** Hongwei Bai, Qianqian Cao, Subang An

**Affiliations:** 1 School of Environment and Spatial Informatics, China University Mining and Technology, Xuzhou, 221116 Jiangsu China; 2 School of Environment and Surveying Engineering, Suzhou University, Suzhou, 234000 Anhui China; 3 School of Mathematics and Statistics, Suzhou University, Suzhou, 234000 Anhui China

**Keywords:** Computer science, Information technology, Scientific data

## Abstract

Satellite clock bias is the key factor affecting the accuracy of the single point positioning of a global navigation satellite system. The traditional model back propagation (BP) neural network is prone to local optimum problems. This paper presents a prediction model and algorithm for the clock bias of the BP neural network based on the optimization of the mind evolutionary algorithm (MEA), which is used to optimize the initial weights and thresholds of the BP neural network. The accuracy of the comparison between clock bias data is verified with and without one-time difference processing. Compared with grey model (GM (1,1)) and BP neural network, this paper discusses the advantages and general applicability of this method from different constellation satellites, different atomic clock type satellites, and the amount of modeling data. The accuracy of the grey model (GM(1,1)), BP, and MEA-BP models for satellite clock bias prediction is analyzed and the root mean square error, range difference error, and the mean of the clock bias data compared. The results demonstrate that the prediction accuracy of the three satellites significantly increased after one-time difference processing and that they have good stability. The prediction accuracy of four sessions of 2 h, 3 h, 6 h, and 12 h obtained using the MEA-BP model was better than 0.74, 0.80, 1.12, and 0.87 ns, respectively. The MEA-BP model has a specific degree of improvement in the prediction accuracy of the different sessions. Additionally, the prediction accuracy of different models has a specific relationship with the length of the original modeling sequence, of which BP model is the most affected, and MEABP is relatively less affected by the length of the modeling sequence, indicating that the MEA-BP model has strong anti-interference ability.

## Introduction

The global navigation satellite system (GNSS) is a wireless signal propagation-based radio navigation and positioning system that provides navigation, positioning, and timing services^1^. The influence of time bias cannot be ignored among other factors affecting the accuracy of navigation and positioning^2^. The time bias of 1 ns, which corresponds to a 3d m distance bias, significantly affects the positioning accuracy of the navigation system^3^. High-precision time systems are crucial for satisfying users’ needs for centimeter positioning^4^. The accuracy of real-time dynamic positioning is improved because of the prediction of satellite clock bias, which is helpful for obtaining the historical information required by satellite autonomous navigation^5^. To improve the real-time positioning accuracy, the accuracy and timeliness of satellite clock bias data must be urgently resolved.

Several models are currently available for predicting satellite clock bias, including the polynomial model, the grey model (GM)^6^, the quadratic polynomial (QP)^7^ model, the autoregressive moving average model (ARMA)^8^, the spectral analysis model^9^, the kalman filter model^10,11^, the wavelet neural network model^12^, and the empirical mode decomposition-support vector machine (EMD-SVM)^13^. Because the satellite clock is easily affected by its external environment and complex characteristics, the satellite clock bias presents nonlinear characteristics, making it challenging for the linear prediction model to accurately represent the change in bias. However, neural networks are more sensitive to nonlinear problems and can overcome the limitations of conventional models for more accurate predictions. Reference^14^ proposed the genetic algorithm back propagation model (GA-BP). The optimized BP neural network is used to make short-term prediction of BDS clock bias, and the results show that its accuracy is better than that of the BP neural network and GM (1,1) model, which shows the feasibility and effectiveness of genetic algorithm optimizing BP neural network for clock bias prediction. However, the optimization model is not used for medium and long term clock error prediction. Reference^15^ proposed a wavelet neural network model based on one-time difference and found that, compared with IGU-P clock products, the average prediction precision for 6, 12, and 24 h was improved by approximately 13.53, 31.56, and 49.46%, respectively. Reference^16^ proposed that the number of hidden layer nodes of the elm network is adaptively adjusted using the concept of an art network and that the prediction accuracy is better than both the quadratic polynomial model and GM in the prediction of 30 days. Reference^17^ proposed that the average prediction errors of the nonlinear autoregressive model with exogenous input recurrent neural network (NARX) in the 6 h and 24 h of the proposed method for all four clocks are equivalent to 23.2%, 17.9%, 36.6%, 16.6%, 20.3%, and 12.5% of the prediction error than those for the three commonly used models. Reference^18^ proposed that the improved back propagation (BP) neural network optimized by heterogeneous comprehensive learning and the dynamic multi-swarm particle swarm optimizer (HPSO-BP) model and its prediction performance is superior to traditional models. Compared with the traditional linear polynomial (LP) model, QP model, GM (1,1) model and ARMA model, the prediction precision can be improved by more than 80%. Reference^19^ used the wavelet neural network to predict the BeiDou satellite clock offset The experimental results show that the prediction accuracy of 6 h can reach 1–2 ns, and the prediction accuracy of 24 h can reach 2–4.6 ns, which is better than the traditional quadratic polynomial model and grey model. However, the improper selection of wavelet basis function in the network will affect the prediction accuracy, and the initial parameters of the wavelet neural network are randomly selected. Reference^20^ constructed a combined prediction model of grey model and BP neural network model to predict satellite clock bias, and the prediction result was better than that of two single models. However, the BP neural network has shortcomings such as slow convergence speed and easy falling into local optimum, which affects the final convergence accuracy.

Although the BP neural network has good adaptability, robustness, and associative memory function, it easily falls into local optimization during the process, and the convergence speed is slow, which affects the final convergence accuracy^21,22^. Because of the characteristics of satellite clock bias and the limitations of the BP neural network, the good global search ability and strong convergence of the mind evolutionary algorithm (MEA) are used. Reference^23^ proposed a BP neural network clock bias prediction model and algorithm optimized by MEA. The prediction performance of the new model is better than that of the three traditional models. However, there is no in-depth study on different consistency satellites, different atomic clock type satellites, or the amount of modeling data. In this study, the MEA is introduced to optimize the weight and threshold value required by the BP neural network, and a clock bias prediction model based on MEA-BP neural network is established. The satellite clock bias data are processed using a one time difference, after which the data are used for modeling and the advantages and general application of this method from different construction satellites, different atomic clock type satellites, and the amount of modeling data are discussed. Following this, the new one time difference is predicted, and the one-time difference is restored to obtain the predicted clock bias data.

## Materials and methods 

### One time difference processing

In the two adjacent epochs before and after, the same satellite’s clock bias data vary slightly, indicating an overall linear trend. The BP neural network is well suited for use with nonlinear data and is not sensitive to the original clock bias data sequence. As the prediction accuracy will be impacted when the BP neural network forecasts the unprocessed data, this study put the original clock bias data through difference processing and transformed the phase data into frequency data. These processed data have good nonlinear characteristics and are suitable for neural network modeling and prediction. A set of n-dimensional satellite clock bias sequences is defined as follows:

1 $$ X = \left\{ {x\left( 1 \right),x\left( 2 \right),x\left( 3 \right), \ldots x\left( {\text{n}} \right)} \right\}, $$

where $x\left( i \right),i = 1,2,3, \ldots n$ represents the clock bias data of the different epochs. By making a difference between the data of adjacent epochs, a new one time difference clock bias data sequence is obtained. This sequence is defined as follows:

2 $$ \Delta X = \left\{ {\Delta x\left( 1 \right),\Delta x\left( 2 \right),\Delta x\left( 3 \right), \ldots \Delta x\left( {n - 1} \right)} \right\} $$

Among $$\Delta x\left( i \right) = x\left( {i + 1} \right) - x\left( i \right)$$.

### GM (1,1) model

Create the differential equation of the GM(1,1) model as follows:

Set the original number sequence as $X^{0} = [x^{0} (1),x^{0} (2), \ldots ,x^{0} (n)]$. Under the initial conditions, the approximate solution of the differential equation can be obtained as

$$ \hat{x}^{(1)} \left( {k + {1}} \right) = \left[ {x^{(0)} \left( 1 \right) - \frac{b}{a}} \right] e^{ - ak} + \frac{b}{a},k = 1,2, \ldots ,n,\quad {\text{the predicted value of}}\quad \hat{x}^{(0)} (k + 1)\quad {\text{is}} $$

3 $$ \hat{x}^{(0)} (k + 1) = \hat{x}^{(1)} (k + 1) - \hat{x}^{(1)} (k) = (1 - e^{a} )[x{}^{(0)}(1) - \frac{u}{a}]e^{ - ak} $$

Among $$k = 1,2, \ldots ,n$$.

Parameter estimation $\hat{b} = \left[ {\hat{a}} \right.$ $\left. {\hat{u}} \right]^{T}$ can be solved by the least square principle, and the satellite clock bias of the prediction epoch can be obtained by substituting the obtained parameters into the above Eq. (3). However, the GM(1,1) is vulnerable to the influence of exponential parameters. As it will enter local optimization, the accuracy of the satellite clock bias is poor when solving the parameters using the least square method.

### BP neural network model

**Figure 1:**
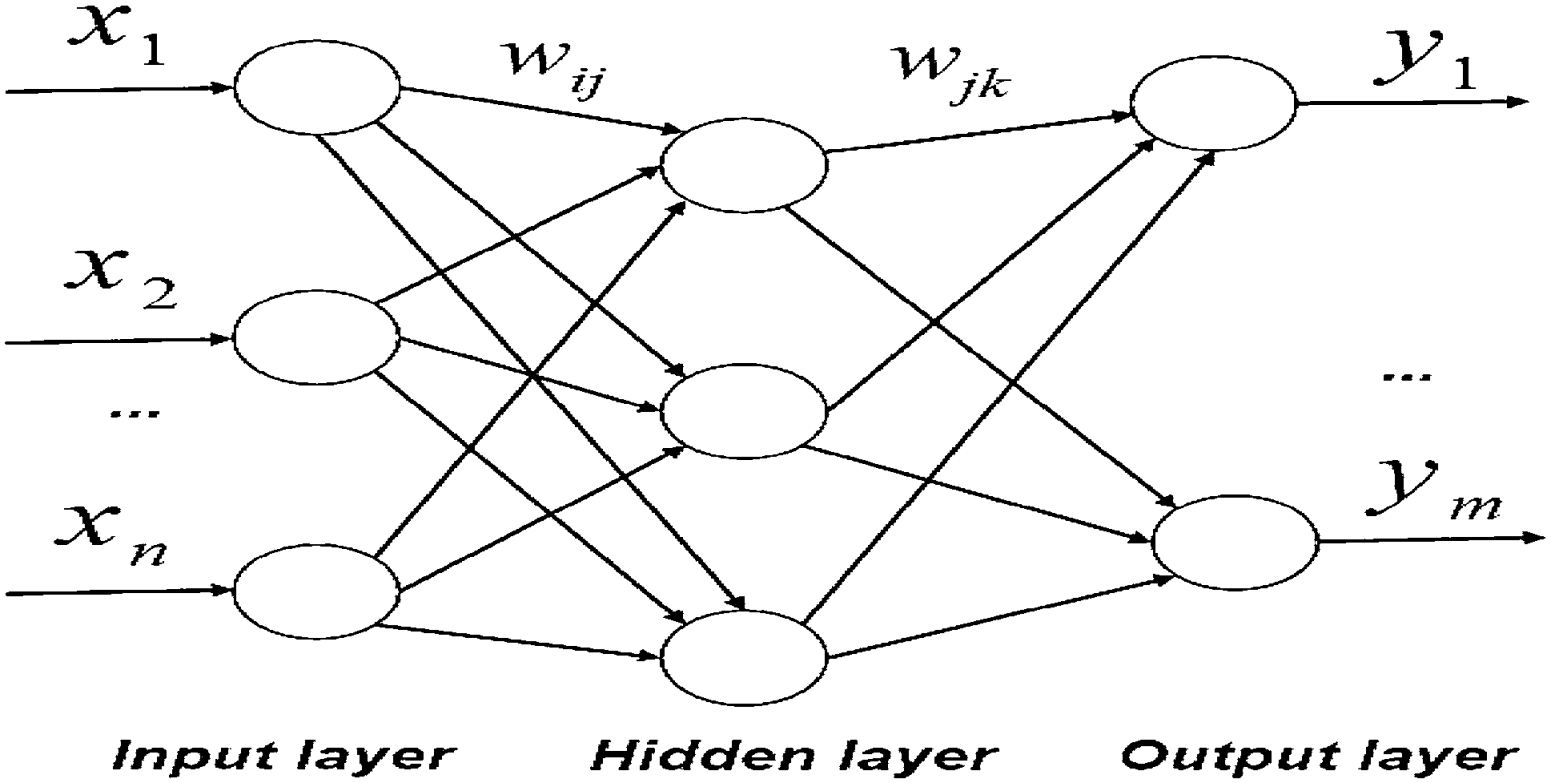
BP neural network topology structure diagram.

A BP neural network is a multilayer feedforward neural network with error back propagation, including input, hidden, and output layers. The neuron state of each layer only affects the neuron state of the next layer^24^. If the output layer cannot obtain the expected output, it will switch to back propagation and adjust the network weight and threshold value according to the prediction bias, so that the prediction output of the BP neural network continues to approach the expected output^25^. The topology of the BP neural network is shown in Fig. 1.

In Fig. 1,$x_{1} ,x_{2} , \ldots ,x_{n}$ are the input values of the BP neural network.$y_{1} ,y_{2} , \ldots ,y_{m}$ are the prediction values of the BP neural network, and $w_{ij}$ and $w_{jk}$ are the weights of the BP neural network.

The hidden layer of the BP neural network can have multiple layers, and the hidden layer is set as five layers in this study. During the training process, calculate hidden layer output $H$ as follows:

4 $$ H_{j} = f \left(\sum\nolimits_{i = 1}^{n} {w_{ij} } x_{i} - a_{j} \right),j = 1,2, \ldots ,5, $$

where $l$ is the number of hidden nodes, $f$ is the hidden layer activation function, and the activation function adopts sigmoid function.

5 $$ f\left( x \right) = \frac{1}{{1 + e^{ - x} }} $$

According to the output $H$ of the hidden layer, connect the weight value $w_{jk}$ and the threshold value $b$, and calculate the predicted output $O$ of the BP neural network.

6 $$ O_{k} = \sum\limits_{j = 1}^{l} {H_{j} } w_{jk} - b_{k} ,k = 1,2, \ldots ,m $$

Calculate network prediction error according to network prediction output $O$ and expected output $Y$.

7 $$ e_{k} = Y_{k} - O_{k} ,k = 1,2, \ldots ,m $$

Update the network connection weights, $w_{ij}$ and $w_{jk}$, according to the network prediction error $e$.

8 $$ w_{ij} = w_{ij} + \eta H_{j} (1 - H_{j} )x(i)\sum\limits_{k = 1}^{m} {w_{jk} } e_{k} ,i = 1,2, \ldots ,n;j = 1,2, \ldots ,l $$

9 $$ w_{jk} = w_{jk} + \eta H_{j} e_{k} ,j = 1,2, \ldots ,l;k = 1,2, \ldots ,m $$

where $\eta$ is the learning rate.

Update network node thresholds $a$ and $b$ according to the network prediction error $e$.

10 $$ a_{j} = a_{j} + \eta H_{j} (1 - H_{j} )\sum\limits_{k = 1}^{m} {w_{jk} } e_{k} ,j = 1,2, \ldots ,l $$

11 $$ b_{k} = b_{k} + e_{k} ,k = 1,2, \ldots ,m $$

### Mind evolutionary algorithm

Chengyi et al. 1998 proposed the MEA, which follows the "population," "individual," and "environment" of the genetic algorithm, and advances the concepts of "convergence" and "alienation." The MEA algorithm is a learning method that is optimized through iteration where every individual in every generation of the evolutionary process becomes a group. A group is divided into several subgroups, and each subgroup includes two types: winning and temporary.

**Figure 2:**
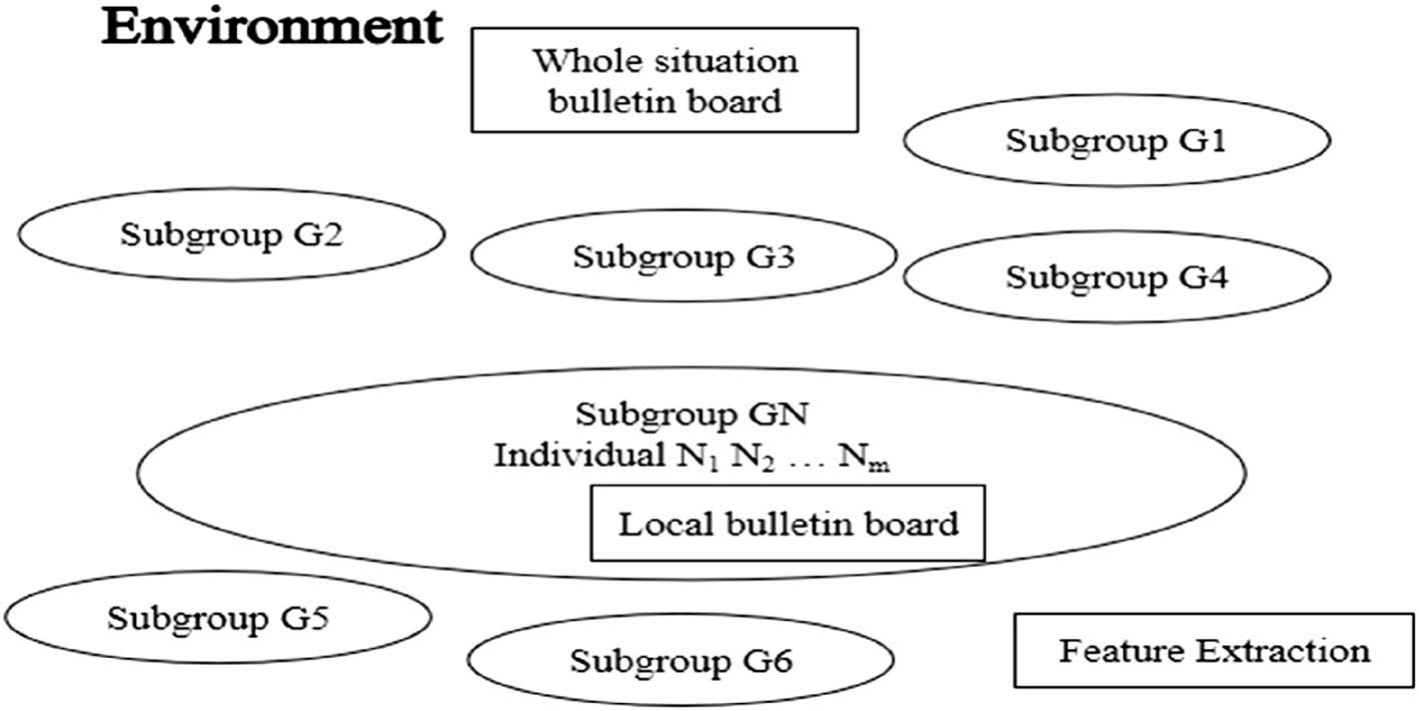
MEA algorithm structure.

The MEA has a faster training speed than genetic algorithms, which significantly reduces the training time of neural networks and is thus more practical. First, the individuals in the subgroups are optimized by the convergence operation, and then the mature subgroups compete globally through the alienation operation, which significantly improves the efficiency of optimization^12^. The structure of the MEA is shown in Fig. 2.

### Clock bias prediction algorithm based on MEA-BP

The BP neural network is a multilayer feedforward neural network. The main characteristics of the BP are signal forward transmission and bias back propagation. Through repeated training, the network weight and threshold are adjusted according to the prediction bias, resulting in a prediction output that is close to the expected output. However, the selection of the initial weight and threshold significantly affects the convergence and accuracy of the BP neural network, and the outcome is prone to local optimization. This study uses the MEA to optimize the weight and threshold in light of the abnormal results of the BP neural network algorithm, which can prevent the BP neural network from entering a local minimum and improve the prediction accuracy of the satellite clock bias.

**Figure 3:**
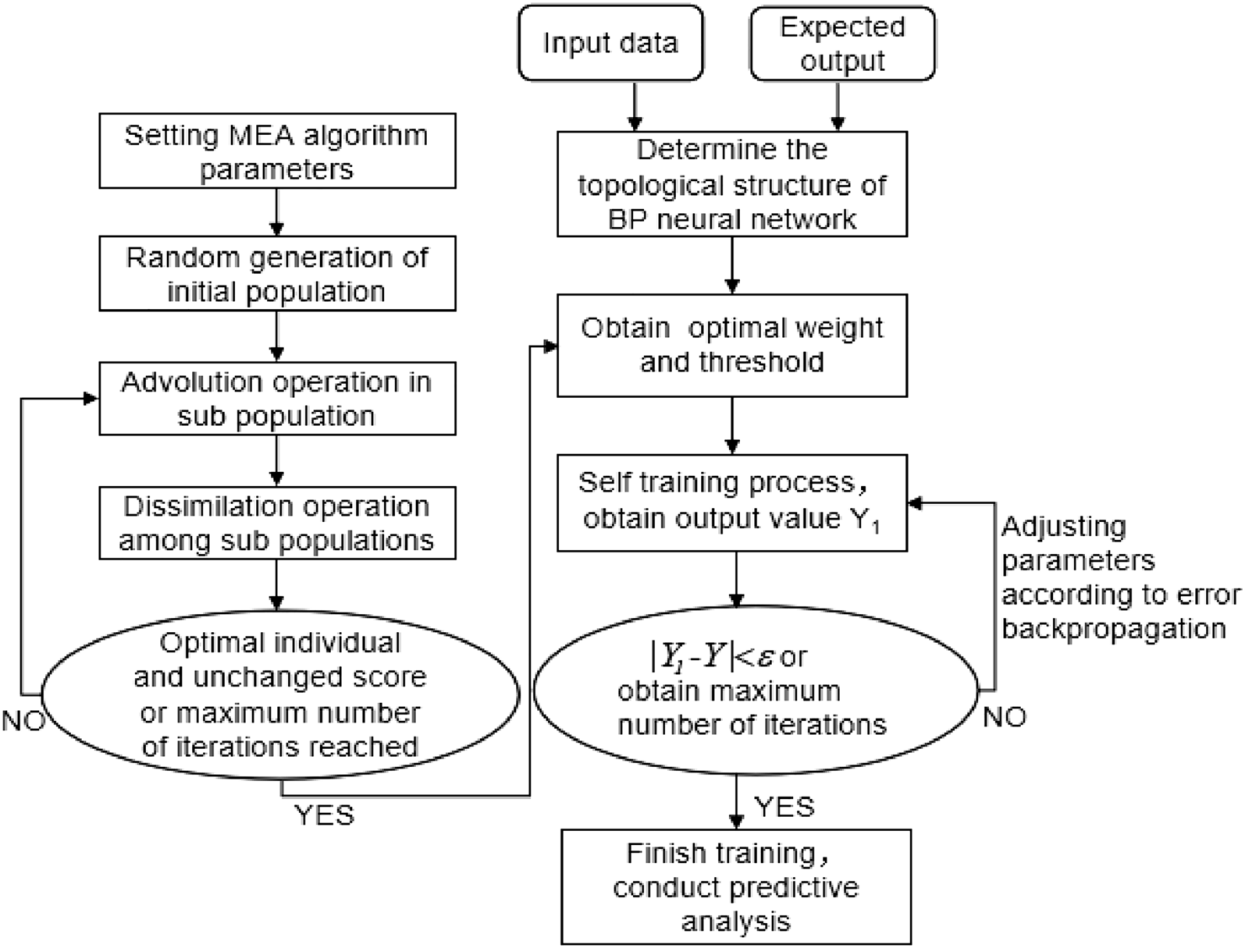
MEA-BP algorithm.

The satellite clock bias data are set as $\left\{ {x_{1} ,x_{2} ,x_{3} , \ldots ,x_{n} } \right\}$, using the N times data to model and predict the clock bias at the next time. To achieve multi calendar satellite clock bias prediction, the sliding window concept is used, where new prediction data are continuously used to replace the previous known data on the basis of ensuring the same number of samples. Figure 3 depicts the MEA-BP algorithm flow.

## Results

To confirm the practicability of the algorithm, the multi-day GPS precision clock bias product data from the international gnss service (IGS) data center are selected for experimental analysis. Because of limited space, this study only lists the satellite clock bias data of GPS system week 2167 on the fourth day (corresponding to July 22, 2021), and the sampling interval is 30 s. In this study, ten satellites, PRN01, PRN02, PRN03, PRN04, PRN10, PRN17, PRN24, C01, C07 and C13 are selected, and the data of the first 12 h are used to model how the satellite clock biases in the next 2 h, 3 h, 6 h, and 12 h. By comparing the predicted clock difference with the real value released from the IGS, the root mean squares error (RMS) and the range difference and mean value of bias are determined using Eqs. (12–14), and the accuracy of prediction results is analyzed^18^.

12 $$ RMS = \sqrt {\frac{{\sum\nolimits_{i = 1}^{n} {\left( {x_{pre,i} - x_{igs,i} } \right)^{2} } }}{n}} $$

13 $$ Range = x_{pre\max } - x_{\begin{subarray}{l} pre\min \\ \end{subarray} } $$

14 $$ Mean = \frac{1}{n}\sum\nolimits_{i}^{n} {\left( {x_{pre,i} - x_{igs,i} } \right)} $$

where $x_{pre,i}$ represents the prediction clock bias of i time model,$x_{igs,i}$ represents the i time real clock released by IGS, n represents the number of clock bias predicted, $x_{pre\max }$ represents the maximum value of on bias, and $x_{pre\min }$ represents the minimum value of prediction bias.

The modeling uses 12 h data with a total of 1440 data and a sampling interval of 30 s for satellite clock bias data. The MEA algorithm has a population size of 1440, and there are five superior and temporary sub populations. For the algorithm to fully find the optimal individual, the number of iterations is set to 100. As the hidden layer setting lacks a theoretical foundation, this study sets the input layer to two (epoch and corresponding clock bias). The number of hidden layer elements is selected with reference to the Kolmogorov theorem.

15 $$ M = N \times 2 + 1, $$

where $M$ and $N$ represent the number of hidden and input layers, respectively.

In this study, the input layer node is set to two (epoch and clock bias). The output layer node is set to one, and the hidden layer node is calculated as three according to Eq. (15); hence, the BP network structure is 2–5–1.

### Test 1

**Figure 4:**
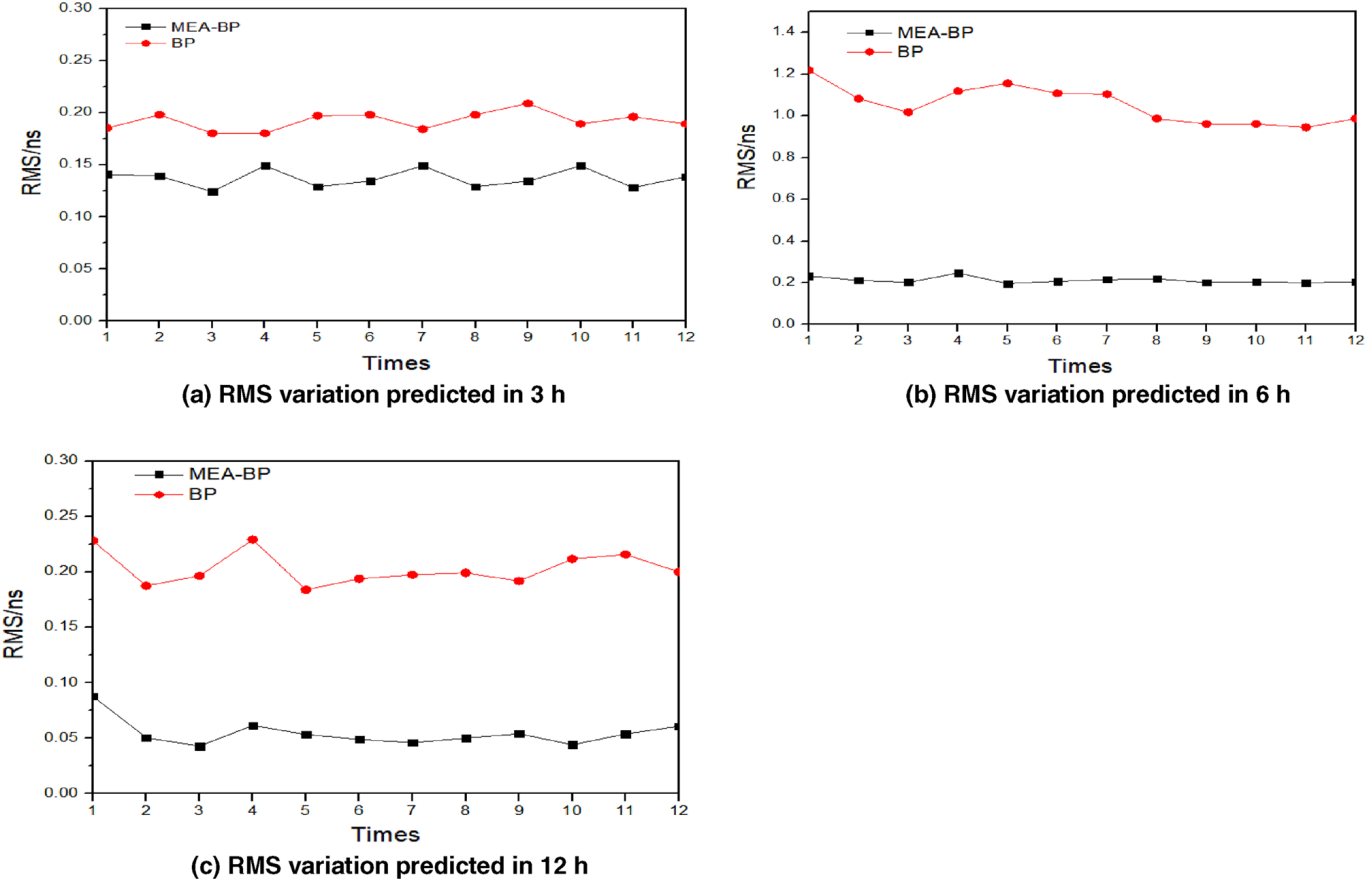
Variation in RMS using MEA-BP and BP to predict for 10 times.

Both the BP and MEA-BP models are compared for their ability to predict satellite clock bias with accuracy. Satellite 10 (other satellites) is selected as an example. To fully compare the prediction effect, the clock bias data of the first 12 h of the day are used to predict the clock bias of the 12 times in the following 3 h, 6 h, and 12 h, respectively. In this test, the BP and MEA-BP models are used to predict the three sessions. Figure 4 shows that the accuracy of multiple forecasts does not vary significantly, demonstrating the viability and stability of the two model’s network structures. During the three sessions, the accuracy of the MEA-BP was better than that of the BP model. This demonstrates that the MEA can optimize the initial weight and threshold of the BP neural network, preventing the BP neural network model from falling into local optimization. Additionally, this effectively improves the prediction accuracy. In general, this shows that the MEA-BP model is reliable for clock bias prediction and is a relatively stable prediction model.

### Test 2

**Figure 5:**
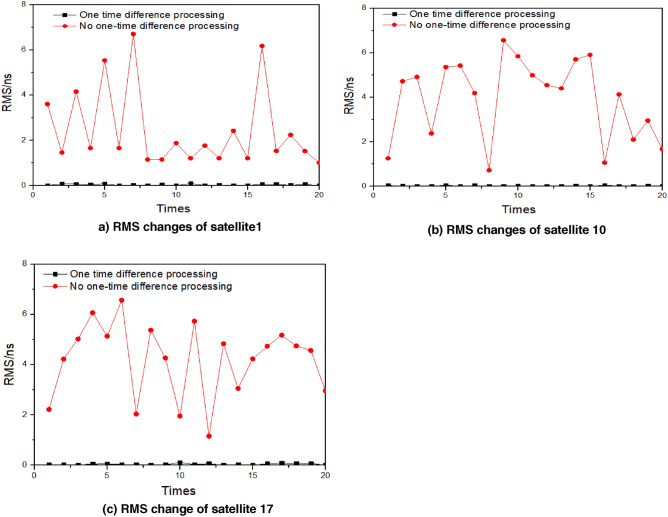
The variation in RMS using BP to predict clock bias before and after one difference about 20 times.

The accuracy of the comparison between clock bias data is verified with and without one-time difference processing. For a neural network, the prediction accuracy increases with the degree of data nonlinearity. The GM must accumulate and reduce the data because it is difficult to provide a qualitative conclusion on the impact of one-time difference processing on the model. Three satellites were randomly selected for prediction: satellites 1, 10, and 17. These satellites use the clock bias data of 12 h before the training day and forecast the clock bias in the next 2 h. The unoptimized BP neural network model is used to predict 20 times, and the changes in the RMS value are compared, as shown in Fig. 5.

According to Fig. 5, when the data without one time difference processing is modeled, the RMS values of satellites 1, 10, and 17 change significantly, and the accuracy is poor. The prediction accuracy of the three satellites significantly improved after one-time difference processing, and the 20-time prediction accuracy was equivalent and had good stability. In conclusion, the BP neural network model uses the one-time difference processing method, which significantly improves the prediction accuracy and stability and is suitable for the network structure used in this study. The following experiments were conducted on the basis of one-time difference.

### Test 3

**Figure 6:**
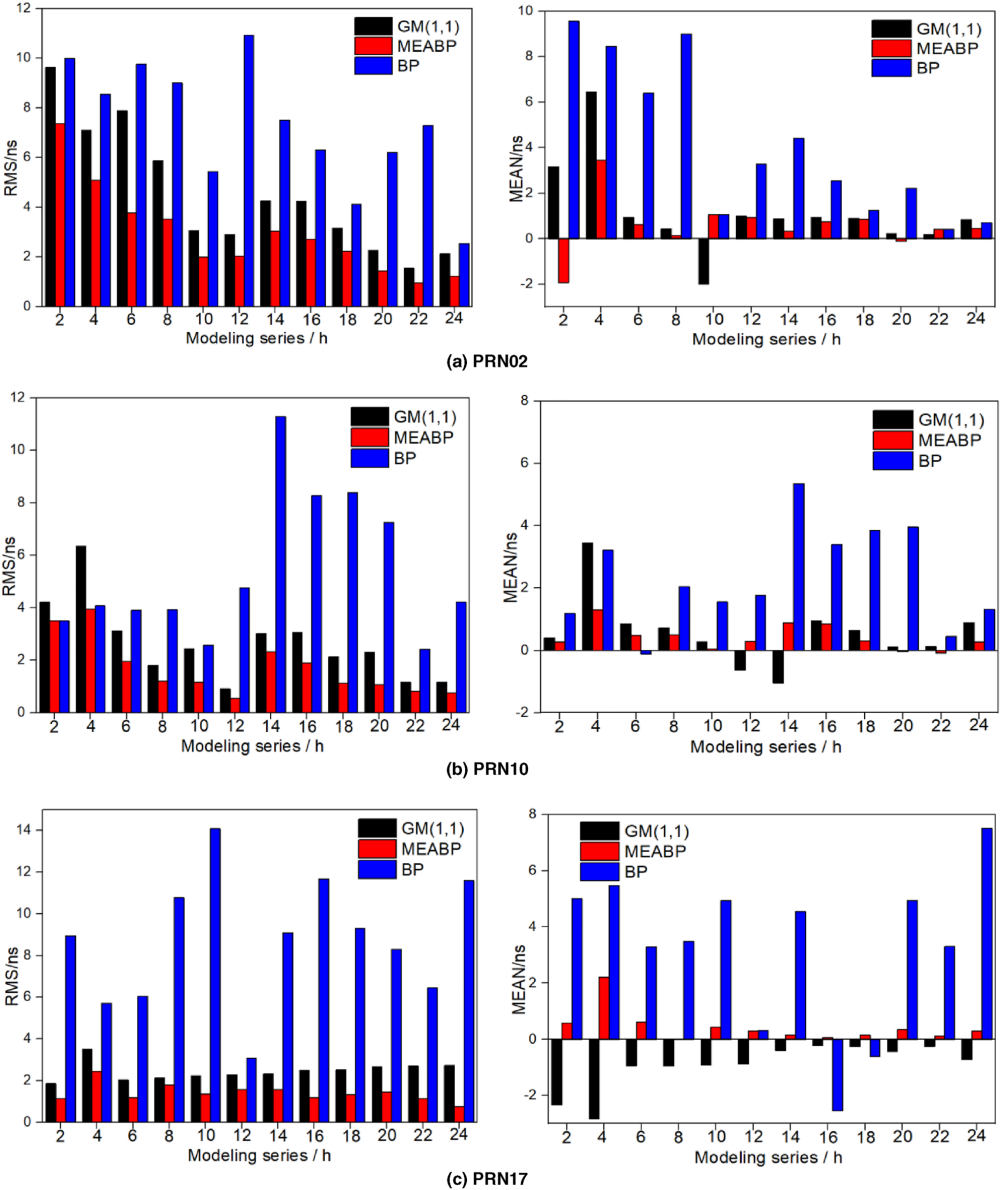
Modeling and prediction accuracy statistics of clock bias sequences with different lengths of three satellites.

To analyze the impact of modeling sequence length on clock bias prediction accuracy, GM (1,1) model, BP model and MEA-BP model were built, respectively, from the clock error sequence of 2 h–24 h of the previous day to predict the clock bias of PRN02, PRN10, and PRN17 satellites in the next 24 h, and the prediction was repeated for 20 times, with the mean value and root mean squares error as the accuracy evaluation index. See Fig. 6 for the statistics of the results of satellite prediction using clock difference sequences of different lengths.

The prediction accuracy of different models has a specific relationship with the original modeling sequence. Among them, BP model is the most affected. GM (1,1) and MEA-BP are relatively less affected by the length of the modeling sequence; the models have strong anti-interference ability, and the prediction results are relatively stable. This also shows that the GM (1,1) model and MEA-BP model have a better modeling effect in the case of less data, but the prediction effect of the MEA-BP model is better than the GM (1,1) model. Taking PRN10 as an example, when the modeling sequence length is 2 h, the mean value and root mean square error of prediction error statistics of the GM (1,1) model, BP model and MEA-BP model are 0.399 ns, 1.188 ns, 0.278 ns and 4.211 ns, and 3.508 ns and 3.5 ns, respectively. When the modeling sequence length is 24 h, the mean value and root mean square error of prediction error statistics of the GM (1,1) model, BP model, and MEA-BP model are 0.892 ns and 1.325 ns, 0.275 ns and 1.155 ns, and 4.213 ns and 0.755 ns, respectively. Compared with the prediction results of 2 h modeling sequence, the prediction accuracy of the GM (1,1) model is increased by 72.57%; the prediction accuracy of the BP model is decreased by 20.10%, and the prediction accuracy of the MEA-BP model is increased by 78.43%. In addition, the precision of the MEA-BP model is 0.22% and 81.82% higher than that of the BP model, respectively, for the modeling and prediction results of 2 h and 24 h clock difference series, and the precision of the MEA-BP model is 16.88% and 34.63% higher than that of the GM (1,1) model, respectively.

### Test 4

The satellites in orbit of the BDS satellite navigation system are divided into three categories according to their orbit types. In this paper, the clock bias data of one satellite is taken as the test data for each type of satellite, and the satellite numbers are C01 (GEO Rb), C07 (IGSO Rb), C13 (MEO Rb), respectively. Data modeling is conducted for the first 12 h of the day to forecast 2 h (240 epochs), 3 h (360 epochs), 6 h (720 epochs), and 12 h (1440 epochs), respectively.

**Figure 7:**
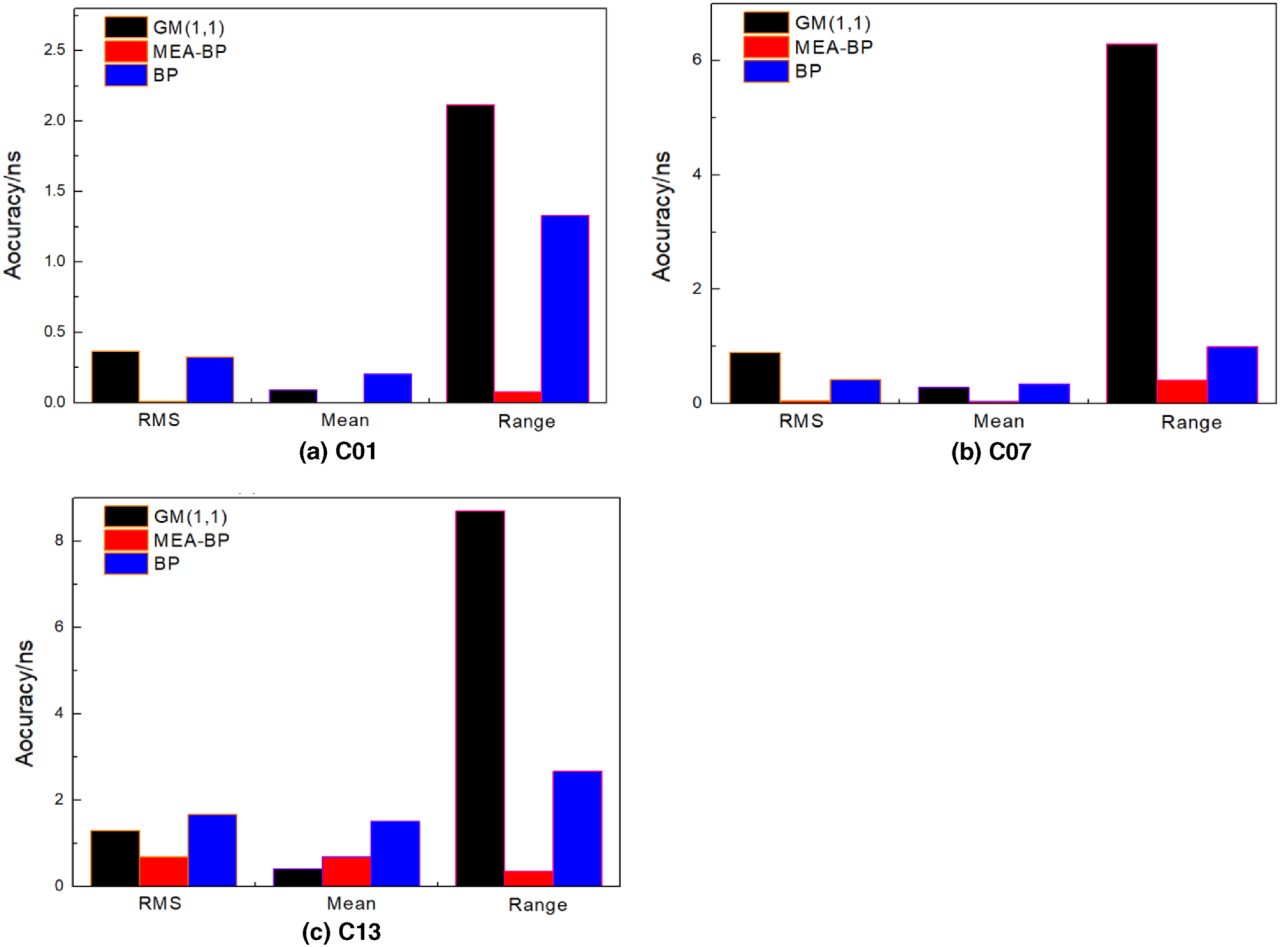
Prediction bar chart of 12 h clock bias of the three satellites.

See Tables 1, 2, 3 and 4 for the specific accuracy comparison of the MEA-BP model and other two models in the prediction of three satellites in the above four sessions. See Fig. 7 prediction bar chart of 12 h clock bias of the three satellites.

**Table 1:** Statistics of 2-h prediction results of three satellites (unit: ns).

Satellite	Statistics	GM(1,1)	BP	MEA-BP
C01	RMS	0.2498	0.2498	0.0055
	MEAN	0.1112	0.1112	0.0054
	RANGE	1.1414	1.1414	0.0059
C07	RMS	0.7802	0.0357	0.0153
	MEAN	0.2502	0.0150	0.0033
	RANGE	4.2040	0.2739	0.1397
C13	RMS	0.9758	1.2991	0.7373
	MEAN	0.1495	0.9280	0.7175
	RANGE	5.4595	4.1746	0.8153

**Table 2:** Statistics of 3-h prediction results of three satellites (unit: ns).

Satellite	Statistics	GM(1,1)	BP	MEA-BP
C01	RMS	0.2535	0.0108	0.0106
	Mean	0.1291	−0.0153	0.0082
	Range	1.1882	0.0267	0.0412
C07	RMS	0.7167	0.0332	0.0267
	Mean	0.2698	0.0243	-0.0258
	Range	4.2040	0.2196	0.0327
C13	RMS	0.9739	1.8642	0.1945
	Mean	0.1686	1.7919	0.1900
	Range	5.4595	2.2839	0.1584

**Table 3:** Statistics of 6-h prediction results of three satellites (unit: ns).

Satellite	Statistics	GM(1,1)	BP	MEA-BP
C01	RMS	0.2581	0.0417	0.0226
	Mean	0.0632	0.0211	0.0019
	Range	1.7869	0.3513	0.1460
C07	RMS	0.8881	0.0697	0.0249
	Mean	−0.1662	0.0323	0.0067
	Range	5.8441	0.8052	0.2848
C13	RMS	1.0995	0.6332	0.3107
	Mean	0.3159	−0.2376	0.3049
	Range	8.5284	5.8388	0.4917

**Table 4:** Statistics of 12-h prediction results of three satellites (unit: ns).

Satellite	Statistics	GM(1,1)	BP	MEA-BP
C01	RMS	0.3646	0.3278	0.0118
	Mean	0.0914	0.2025	0.0005
	Range	2.1165	1.3296	0.0788
C07	RMS	0.8947	0.4222	0.0474
	Mean	0.2816	0.3389	0.0414
	Range	6.2927	0.9945	0.4065
C13	RMS	1.2960	1.6628	0.6868
	Mean	0.4047	1.5038	0.6839
	Range	8.6972	2.6676	0.3528

The prediction accuracy of different models has a specific relationship with the predicted epoch length, among which BP model is the most affected. GM (1,1) and MEA-BP models are relatively less affected by the predicted epoch length; the model has strong anti-interference ability, and the prediction results are relatively stable. The prediction effect of the MEA-BP model is better than that of the GM (1,1) model and BP model. Taking C07 as an example, when the forecast epoch length is 2 h, the root mean squares error of the GM (1,1) model, BP model, and MEA-BP model are 0.7802 ns, 0.0357 ns, and 0.0153 ns, respectively. When the prediction epoch length is 12 h, the root mean squares error of the prediction error statistics of the GM (1,1) model, BP model, and MEA-BP model is 0.8947 ns, 0.4222 ns, and 0.0474 ns, respectively. Compared with the prediction results of the prediction epoch length of 2 h, the prediction accuracy of the GM (1,1) model is reduced by 0.1145 ns. the prediction accuracy of the BP model is reduced by 0.3865 ns, and the prediction accuracy of the MEA-BP model is reduced by 0.0321 ns. In addition, the accuracy of the MEA-BP model is 57.14% and 88.77% higher than that of the BP model, and the accuracy of the MEA-BP model is 98.04% and 94.70% higher than that of the GM (1,1) model, respectively, for the prediction results of 2 h and 12 h epoch length.

### Test 5

**Figure 8:**
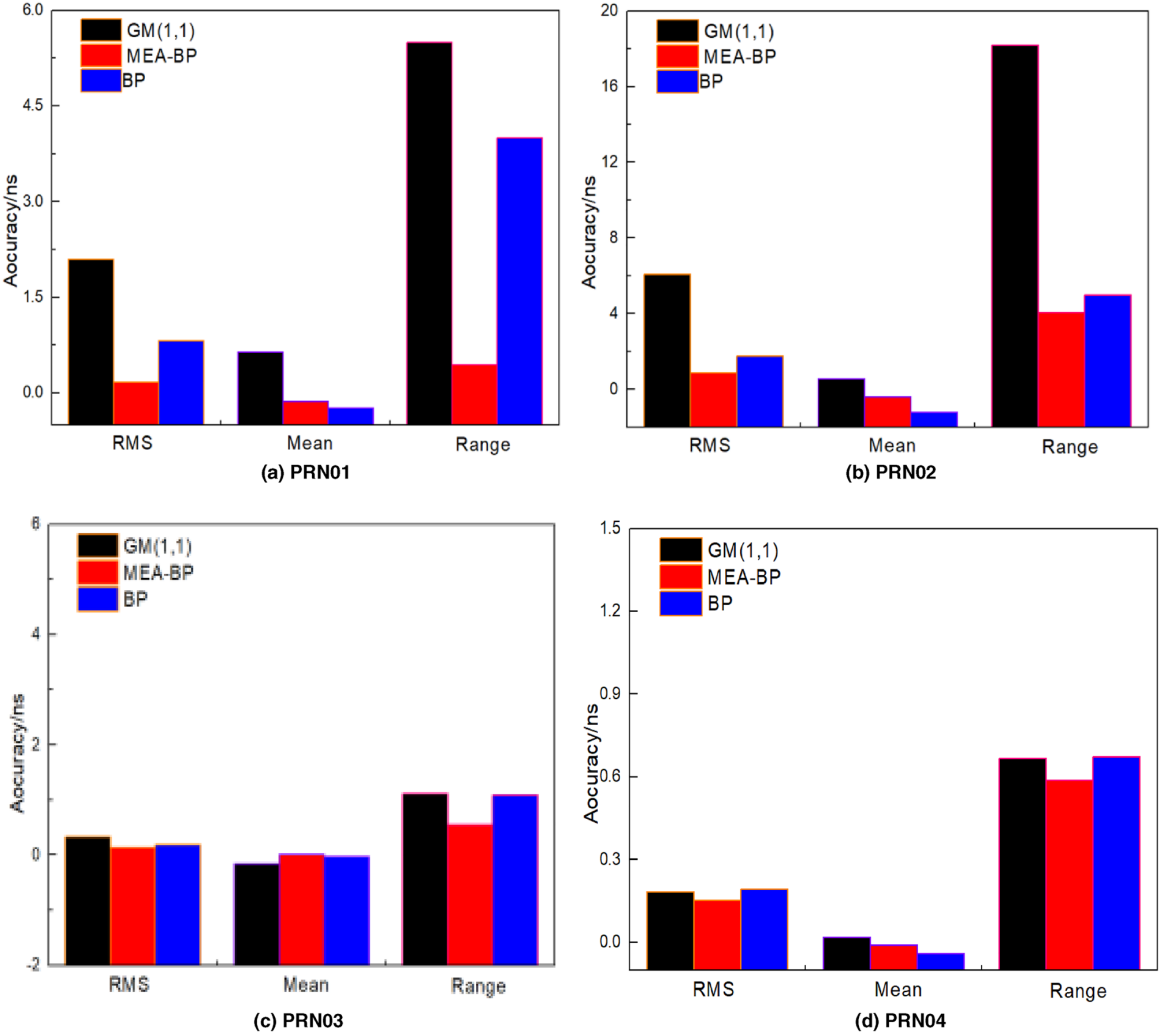

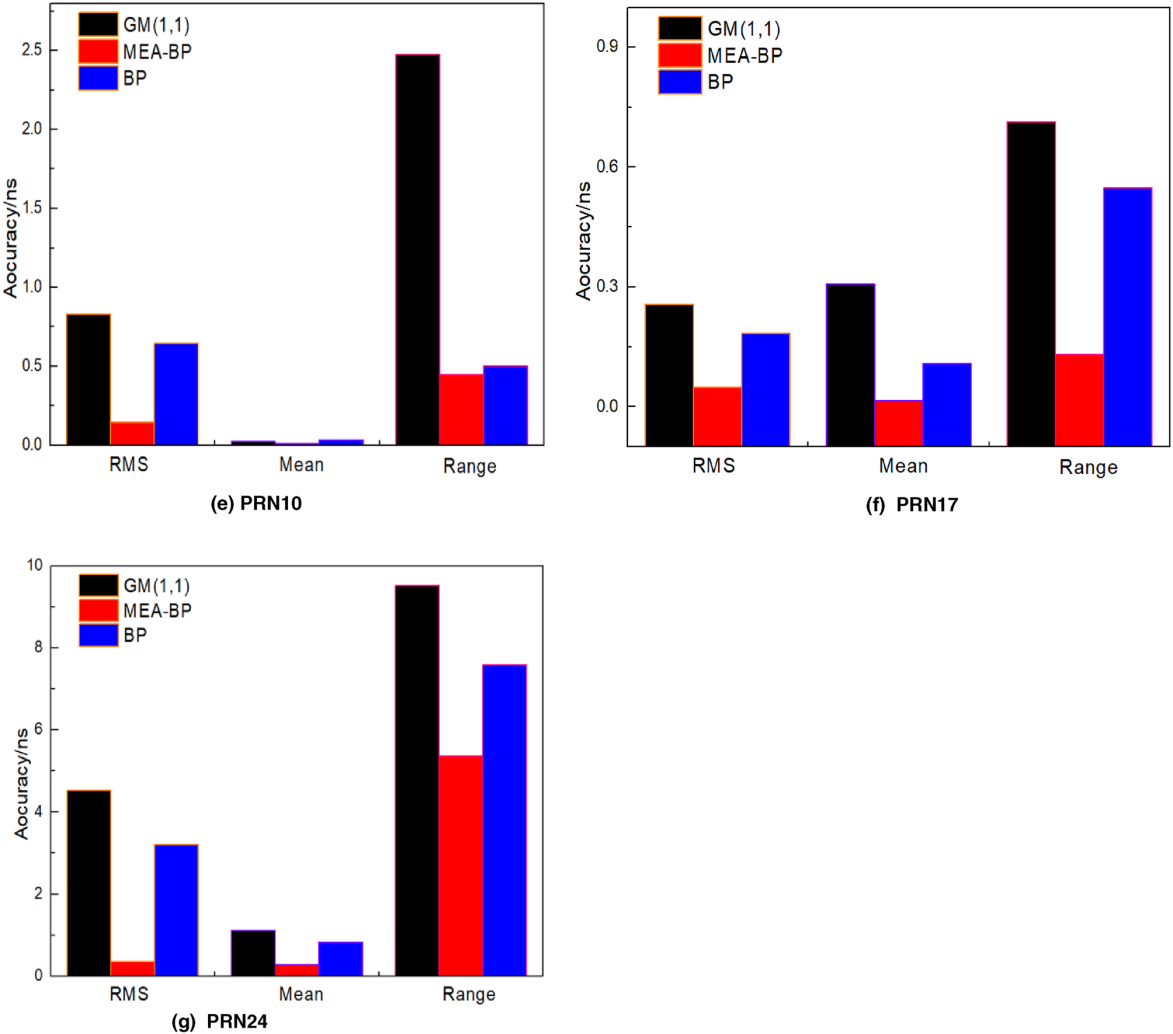
Prediction bar chart of 12 h clock bias of the seven satellites.

**Figure 9:**
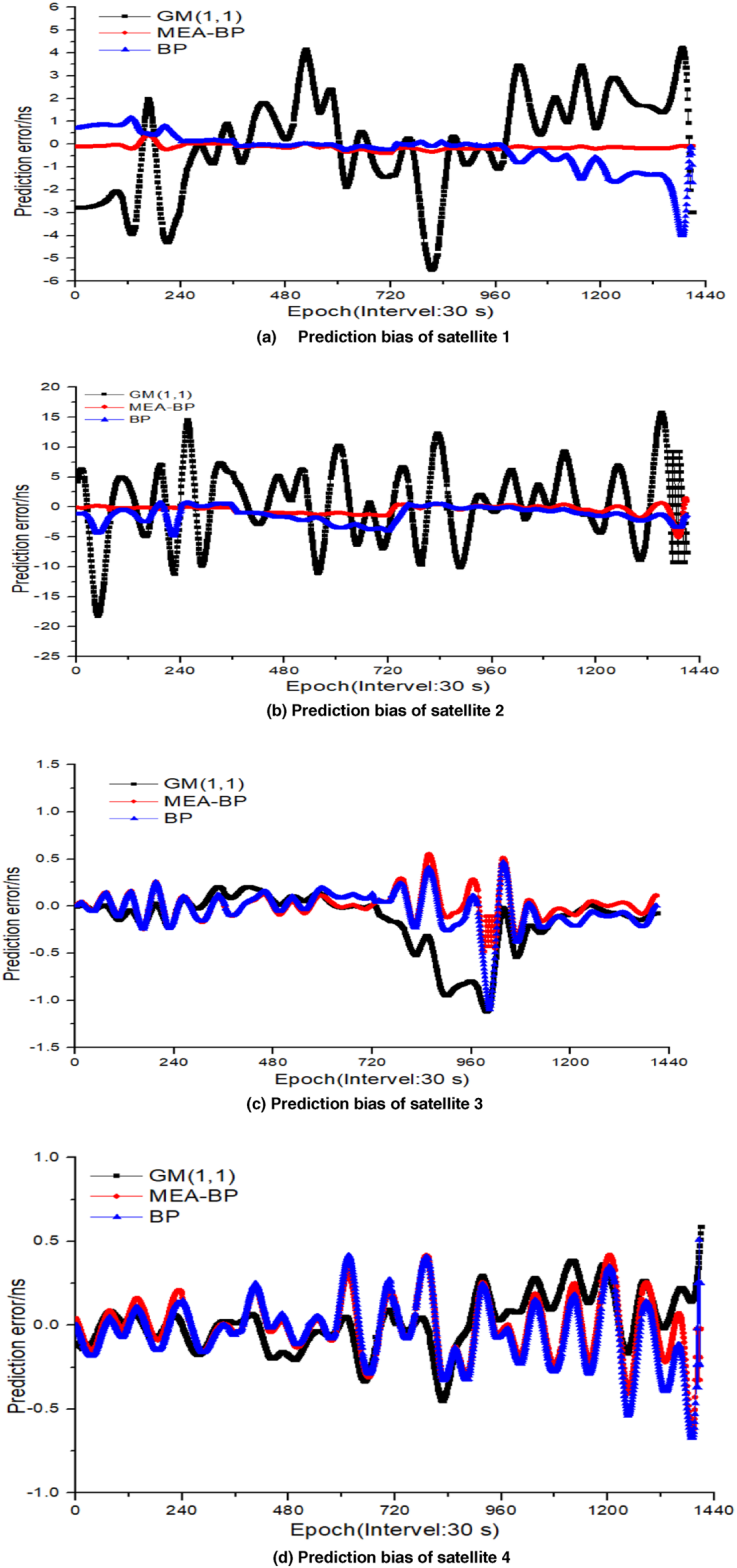

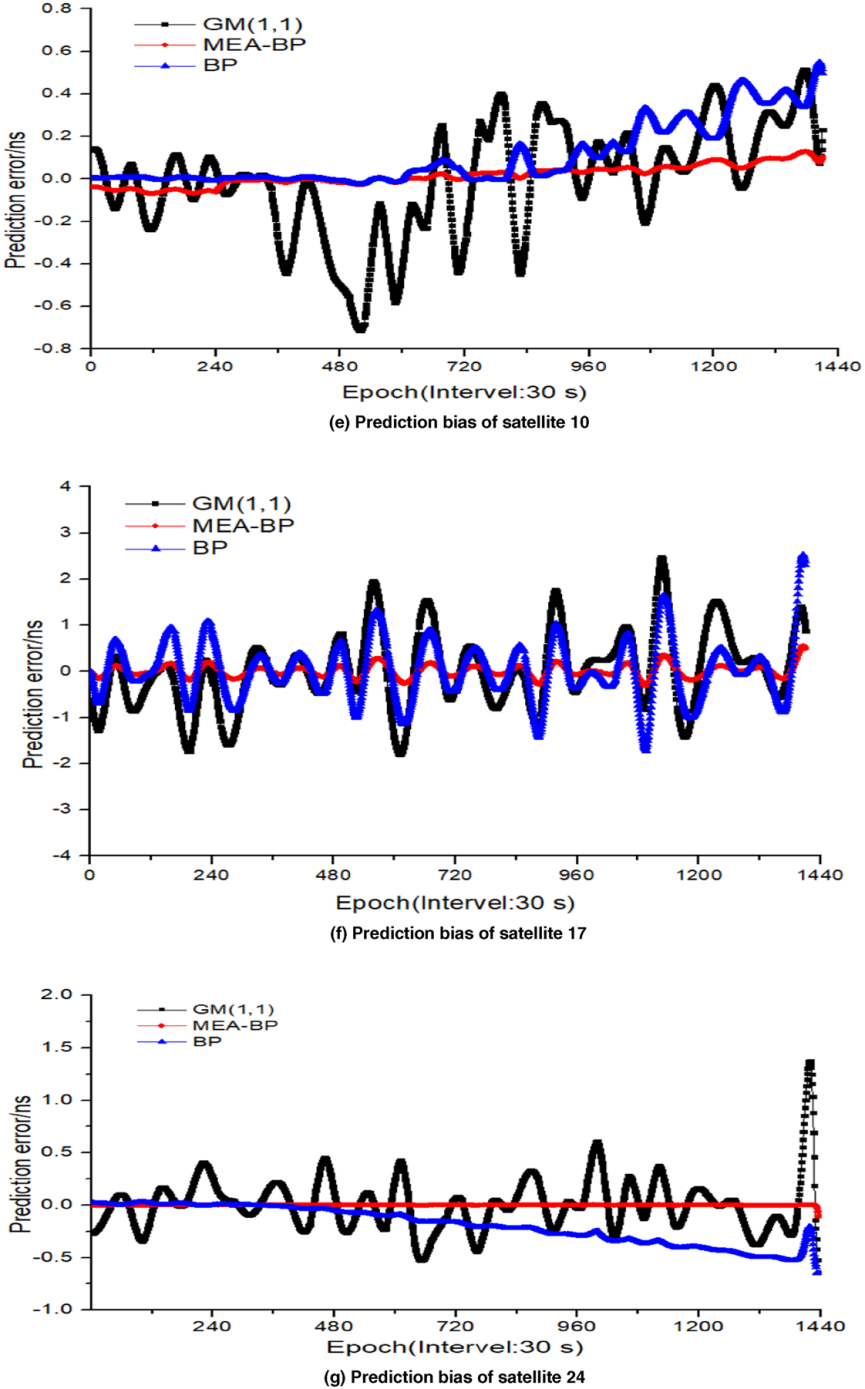
Comparison of the prediction bias of 12 h clock bias of the seven satellites.

The prediction accuracy of the MEA-BP model was compared with the BP and GM (1,1) models. The data of the first 12 h of the day from the PRN01 (Bock IIF Rb) , PRN02 (BockII IIR Rb), PRN03 (Bock IIF Rb), PRN04 (Bock IIA Rb), PRN10 (Bock IIA Cs), PRN17 (Bock IIR-M Rb), and PRN24 (Bock IIF Cs) satellites are modeled to predict 2 h (240 epoch), 3 h (360 epoch), 6 h (720 epoch), and 12 h (1440 epoch), respectively. The accuracy comparison between the MEA-BP model and the other two models in terms of the root mean square bias, mean value, and range difference in the prediction of the seven satellites in the above four sessions is provided in Tables 5, 6, 7 and 8. To achieve multi calendar satellite clock bias prediction, the sliding window concept is used where new prediction data are continuously used to replace the previous known data on the basis of ensuring the same number of samples. The root mean square bias, range difference, and mean value of seven satellites for 12 h prediction bar chart and the MEA-BP model, GM (1,1), and BP model prediction bias trend chart are provided, as shown in Figs. 8 and 9, because the 12 h prediction bias includes the 2 h, 3 h, and 6 h.

**Table 5:** Statistics of 2 h prediction results of the seven satellites (unit: ns).

PRN	Statistics	GM(1,1)	BP	MEA-BP
PRN01	RMS	3.0018	0.8228	0.2013
	Mean	−2.3286	0.7546	−0.0372
	Range	2.1298	3.5254	0.2489
PRN02	RMS	9.6819	3.0877	0.2351
	Mean	−2.0977	−1.8935	−0.1143
	Range	1.0641	3.1666	0.3466
PRN03	RMS	1.4018	0.0571	0.0280
	Mean	−0.7493	−0.0514	−0.0133
	Range	1.3963	0.3089	0.2417
PRN04	RMS	0.6896	0.2665	0.2520
	Mean	−0.3998	−0.2589	0.2433
	Range	4.1886	0.3118	0.2008
PRN10	RMS	1.3856	0.6833	0.3759
	Mean	−0.2000	−0.5988	−0.1651
	Range	1.1923	1.3525	0.6325
PRN17	RMS	9.4869	2.4984	0.6630
	Mean	−5.0606	2.4812	0.6491
	Range	7.9242	3.2889	1.5196
PRN24	RMS	2.1439	0.3719	0.2936
	Mean	0.6510	−0.3839	−0.1920
	Range	3.9153	2.0862	0.3402

**Table 6:** Statistics of 3 h prediction results of the seven satellites (unit: ns).

PRN	Statistics	GM(1,1)	BP	MEA-BP
PRN01	RMS	0.9614	0.1392	0.0350
	Mean	−0.2700	0.1323	0.0345
	Range	8.9918	0.3816	0.0753
PRN02	RMS	1.1075	0.6600	0.3695
	Mean	3.5702	0.4224	−0.0712
	Range	1.1820	0.6105	0.5093
PRN03	RMS	1.2803	0.2353	0.1125
	Mean	0.9405	0.2041	0.1110
	Range	5.5855	0.4628	0.1343
PRN04	RMS	0.8235	0.1098	0.0634
	Mean	−0.2840	−0.0855	−0.0572
	Range	2.6261	0.2376	0.0994
PRN10	RMS	0.9397	0.1866	0.1497
	Mean	−0.2700	−0.0986	−0.0324
	Range	8.0174	2.2130	1.5479
PRN17	RMS	6.9569	0.8423	0.7995
	Mean	−2.7411	−0.7716	−0.7572
	Range	2.4086	1.2315	0.7922
PRN24	RMS	1.9023	1.0677	0.1855
	Mean	0.6506	−0.5762	0.1188
	Range	4.9153	2.6862	1.6584

**Table 7:** Statistics of 6 h prediction results of the seven satellites (unit: ns).

PRN	Statistics	GM(1,1)	BP	MEA-BP
PRN01	RMS	1.8793	0.3355	0.2145
	Mean	0.6771	−0.1649	−0.1103
	Range	7.5031	5.7073	1.5068
PRN02	RMS	6.1156	2.5032	1.1199
	Mean	1.1964	−2.3045	−1.1037
	Range	4.2312	4.0006	1.7239
PRN03	RMS	1.0474	0.7156	0.0540
	Mean	0.7442	0.6068	0.0436
	Range	4.7690	1.3572	0.3245
PRN04	RMS	1.4837	0.3657	0.1388
	Mean	−0.8993	0.3228	0.0171
	Range	5.8229	1.5393	0.8765
PRN10	RMS	4.1697	1.0338	0.2306
	Mean	−2.9248	0.6100	0.0762
	Range	3.7066	3.5804	2.6640
PRN17	RMS	6.7947	2.5631	0.8993
	Mean	1.6648	0.9424	0.2740
	Range	2.5075	1.7186	0.8284
PRN24	RMS	2.5002	1.6695	0.3364
	Mean	1.1004	0.6330	0.3217
	Range	5.0064	4.4395	1.7386

**Table 8:** Statistics of 12 h prediction results of the seven satellites (unit: ns).

PRN	Statistics	GM(1,1)	BP	MEA-BP
PRN01	RMS	2.1009	0.8243	0.1757
	Mean	0.6464	−0.2378	−0.1300
	Range	5.4990	4.0022	0.4453
PRN02	RMS	6.0756	1.7608	0.8648
	Mean	0.5666	−1.2037	−0.4135
	Range	18.1817	4.9803	4.0806
PRN03	RMS	0.3298	0.1944	0.1418
	Mean	−0.1483	−0.0310	0.0092
	Range	1.1156	1.0985	0.5536
PRN04	RMS	0.1844	0.1940	0.1541
	Mean	0.0202	−0.0391	−0.0098
	Range	0.6685	0.6741	0.5885
PRN10	RMS	0.2571	0.18304	0.0500
	Mean	0.3070	0.10810	0.0163
	Range	0.7139	0.5488	0.1299
PRN17	RMS	0.8321	0.6434	0.1432
	Mean	0.0219	0.0313	0.0084
	Range	2.4736	0.5038	0.4487
PRN24	RMS	2.5002	1.6695	0.3364
	Mean	1.1004	0.6330	0.3217
	Range	5.0064		1.7386

## Discussion

Seen from the fluctuation of prediction error of the MEA-BP model algorithm, the error fluctuation amplitude of the rubidium clock (PRN01, PRN02, PRN03, PRN04, PRN17) is within 0.9 ns. For the BP model and GM (1,1) model, the error fluctuation amplitude of rubidium clock is within 6.1 ns, and the error fluctuation amplitude of the cesium clock is within 4.5 ns. In addition, from Tables 5, 6, 7 and 8, it can be seen that the rubidium clock is relatively stable compared with the cesium clock on the whole, but the root mean square error of some satellite prediction results can be seen, The prediction accuracy of the cesium clock using the GM (1,1) model, BP model, and MEA-BP model can be comparable to that of the rubidium clock, and even better than the rubidium clock in some cases. For example, in 12 h prediction, the RMS of cesium clock (PRN24) and MEA-BP model is 0.3576 ns, which is better than the rubidium clock PRN02 satellite, and the gm model is 4.5281 ns, which is better than the rubidium clock PRN02 satellite.

The forecast results demonstrate that of the four sessions shown in Tables 5, 6, 7 and 8, using satellite 1 as an example, the prediction accuracy (RMS) of the MEA-BP at 2, 3, 6, and 12 h is less than 0.22 ns. The MEA-BP model has a specific anti-interference ability and changes less as the prediction time increases. By comparing the mean and RMS values, the accuracy of the MEA-BP model proposed in this study is higher than that of the other two common models in clock bias prediction in different periods. This is particularly evident in 12 h prediction, with minimum and maximum increases of 16% and 91%, respectively. Additionally, as the forecast duration increases, excluding the fact that the forecast accuracy of satellites 2 and 17 is slightly poor, the forecast bias of satellites 2 and 17 is within 1.2 ns and 0.9 ns, respectively, and the rest are controlled within 0.4 ns, indicating that the MEA-BP has good stability. The range value in the table is smaller than that of the BP and GM (1,1) models, indicating that the MEA-BP model has good prediction performance.

The comparison in Fig. 9 shows that the overall change trend of the prediction bias of the MEA-BP and BP neural network models is similar but that the MEA-BP model is relatively stable.

Comparing the prediction bias curves of the MEA-BP and BP neural network models in Fig. 9a,c,e,f, when the BP neural network prediction has a significant bias, the size and direction of the bias can be changed to a certain extent after the optimization of the MEA algorithm, effectively ensuring the prediction accuracy.

Figure 9 shows that, as the prediction epoch increases, the prediction result of the MEA-BP model becomes more stable than that of the BP and GM models. When the bias value is small, there is no divergence, and the 12 h prediction bias of the seven satellites fluctuates around zero. When the prediction time increases, the overall bias fluctuates less, and the predicted clock bias value is more consistent with the real clock bias released by IGS, indicating good stability. This demonstrates that the BP model optimized by the MEA does not fall into local optimization when predicting the increase in epoch, which shows the stability and practicability of the MEA-BP model and the feasibility of the neural network structure to a certain extent.

It can be seen from Fig. 9 that the prediction error of satellite clock bias shows a significant periodic characteristic. This is because of the significant periodic term of satellite clock bias sequence. The periodic term of clock bias is different for satellites of different orbital types, while the periodic term of clock bias is also different for satellites of the same orbital type. The main period of the satellite clock bias data is approximately 1/2 or 1 time of the satellite orbit period. In addition, in the process of simultaneous calculation of satellite orbit and clock bias based on multi satellite joint orbit determination, part of the orbit error is absorbed by the clock bias. In addition to coupling with the orbit period, the satellite clock may also be related to the changes of the external day and night environment. The satellite clock bias results have periodic fluctuations.

## Conclusions

As bias accumulates over time, the common model, which is used for clock bias prediction because of the nonlinear characteristics of satellite clock bias, has unstable accuracy. To address the issues with the BP neural network algorithm in satellite clock bias prediction and obtain better initialization weight, threshold, and other parameters, this study adopts the MEA algorithm. The algorithm improves the accuracy of satellite clock bias modeling, successfully prevents the BP neural network from falling into local optimization, and increases the calculation speed of the BP algorithm. The MEA-BP model, which is suitable for satellite clock bias prediction, is then proposed. The paper discusses the advantages and general applicability of this method from different constellation satellites, different atomic clock type satellites, and the amount of modeling data. By analyzing the prediction accuracy of the three models, the MEA-BP model has good prediction accuracy and stability. The accuracy and bias curves do not vary significantly as the prediction time increases. Compared with the traditional BP and GM models, the lowest accuracy can be improved by 5.44%, 5.08%, 36.07%, and 16.43% at 2, 3, 6, and 12 h, respectively, while the highest accuracy can be improved by 98.00%, 96.36%, 94.84%, and 91.64%, respectively.

The MEA-BP model has strong adaptability, global search ability, and global convergence. Through multiple "convergence" and "alienation" iterative operations, global optimization is performed, and the global optimal solution of the initial weight and threshold in the BP neural network is obtained.

The MEA-BP model outperforms the traditional BP model in terms of prediction accuracy and efficiency. The optimized value is more accurate because of the competitive learning of different subgroups and individuals, which improves the accuracy of the neural network and the prediction accuracy of the clock bias prediction model. The model performs well in the short-term predictions and has a strong real-time performance. As such, the model can be used for high-precision prediction of satellite clock bias.

Many factors affect the accuracy of neural network clock bias prediction such as data sampling interval, the amount of modeling data, and the selection of hidden layer nodes. Additionally, different satellite data have different sequence changes after one time difference processing, necessitating an adjustment to the corresponding network structure, which requires further research.

## Data Availability

The authors thank the IGS Data Center of Wuhan University for providing open source data for this study. The website is http://www.igs.gnsswhu.cn/index.php/home/data_product/igs.html.
